# Integrating meristem initiation and floral fate control: toward *de novo* flower organogenesis *in vitro*

**DOI:** 10.3389/fpls.2026.1739266

**Published:** 2026-02-16

**Authors:** Niels R. Peeters, Jason Gardiner, Marcel Proveniers

**Affiliations:** Translational Plant Biology, Department of Biology, Science4Life, Utrecht University, Utrecht, Netherlands

**Keywords:** *de novo* flower organogenesis (DNFO), developmental regulators (DRs), direct-flowering, meristem fate transition, tissue-culture

## Abstract

Plant tissue culture is a cornerstone of green biotechnology, facilitating key applications such as genetic transformation and clonal propagation. In tissue culture, meristems serve as the source of regenerative growth, enabling complete plants to develop from small explants or single cells. While tissue culture accelerates propagation, inducing flowering remains a critical bottleneck for breeding programs, as flowering typically requires an extended vegetative phase. The ability to induce direct flowering *in vitro*, effectively bypassing the vegetative phase, would decouple reproduction from endogenous developmental timing and environmental cues. We frame this approach as *de novo* flower organogenesis (DNFO), analogous to *de novo* shoot (DNSO) and root organogenesis (DNRO), to conceptualize strategies for directly inducing floral meristems. DNFO could accelerate breeding cycles, streamline the development of transgenic lines, and enhance experimental throughput. Meristems are typically induced via hormone supplementation or ectopic expression of developmental regulators (DRs). However, regenerated plants still undergo sequential fate transitions before floral induction. This transition is mainly controlled by floral pathway integrators (FPIs), such as *FLOWERING LOCUS T* (*FT*) and *SUPPRESSOR OF OVEREXPRESSION OF CONSTANS1* (*SOC1*), and floral meristem identity (FMI) genes, including *LEAFY* (*LFY*) and *APETALA1* (*AP1*). In this review, we examine the potential to simultaneously induce meristems and floral fate to enable DNFO in tissue culture. Although constitutive or early expression of FPIs and FMIs accelerates flowering *in planta* and *in vitro*, it has so far not been sufficient to induce flowers directly from undifferentiated tissue. We propose that this limitation may reflect the absence of UNUSUAL FLORAL ORGANS (UFO), a cofactor of LFY that is required for the full activation of floral identity programs. We hypothesize that spatiotemporally coordinated co-expression of *UFO* and *LFY* could bypass intermediate developmental stages and trigger direct formation of floral meristems, hence flowers, in *in vitro* culture.

## 1 Introduction

Plant tissue culture is a crucial step in green biotechnology, as it enables the production of virus-free plants, facilitates clonal propagation for the mass production of uniform plants from elite genotypes, and supports genetic transformation and somatic embryogenesis (Thorpe, 2007). In standard tissue culture approaches, pluripotent plant tissue (callus) is initiated on callus-inducing medium (CIM). Next, it is transferred to shoot-inducing medium (SIM). Here, a new shoot meristem differentiates and forms a shoot (Skoog and Miller, 1957; Ikeuchi et al., 2019). This shoot is then transferred to a specific rooting medium until it forms roots, at which point the propagule can be transferred to soil. Due to the higher regenerative capacity of juvenile tissues compared to adult tissues, tissue culture protocols often rely on juvenile explants (Zhang et al., 2015; Lee et al., 2020). However, this preference introduces a critical challenge: plants regenerated from juvenile material generally retain a juvenile physiological state and therefore must undergo an extended vegetative growth phase before reaching reproductive competence and initiating flowering. The ability to bypass vegetative development and induce flowering directly from *in vitro* tissue could substantially reduce generation times and enhance breeding efficiency.

However, despite detailed mechanistic insights into floral fate determination and meristem identity transitions (Maple et al., 2024), the direct specification of floral meristems from pluripotent tissue remains a challenge. While the key regulators governing meristem formation, maintenance, and phase transitions are well characterized, their deployment to circumvent the canonical vegetative-to-reproductive transition *in vitro* remains largely unexplored.

In this review, we summarize the current understanding of meristem formation and the developmental processes that guide meristem fate toward flowering, and introduce the concept of *de novo* flower organogenesis (DNFO), analogous to *de novo* shoot (DNSO) and root organogenesis (DNRO). DNFO describes the direct specification and formation of floral meristems and flowers from pluripotent or regenerating tissue, bypassing the canonical vegetative phase. This conceptual framework allows us to discuss strategies by which targeted expression of developmental regulators and flowering genes can enable direct flower induction in tissue culture, potentially accelerating breeding and reducing generation times.

DNFO differs from DNSO/DNRO in that it specifically targets reproductive structures, rather than vegetative shoots or roots. Conceptually, it integrates meristem initiation, maintenance, and floral fate acquisition into a single coordinated process.

## 2 Meristem initiation and maintenance across developmental and contexts

Plant meristems are specialized regions of pluripotent, actively dividing cells that serve as the source of all new organs and tissues in a plant. Their primary role is to generate new cells for growth and organ formation, making them essential for primary growth, branching, and reproduction.

**Figure 1:**
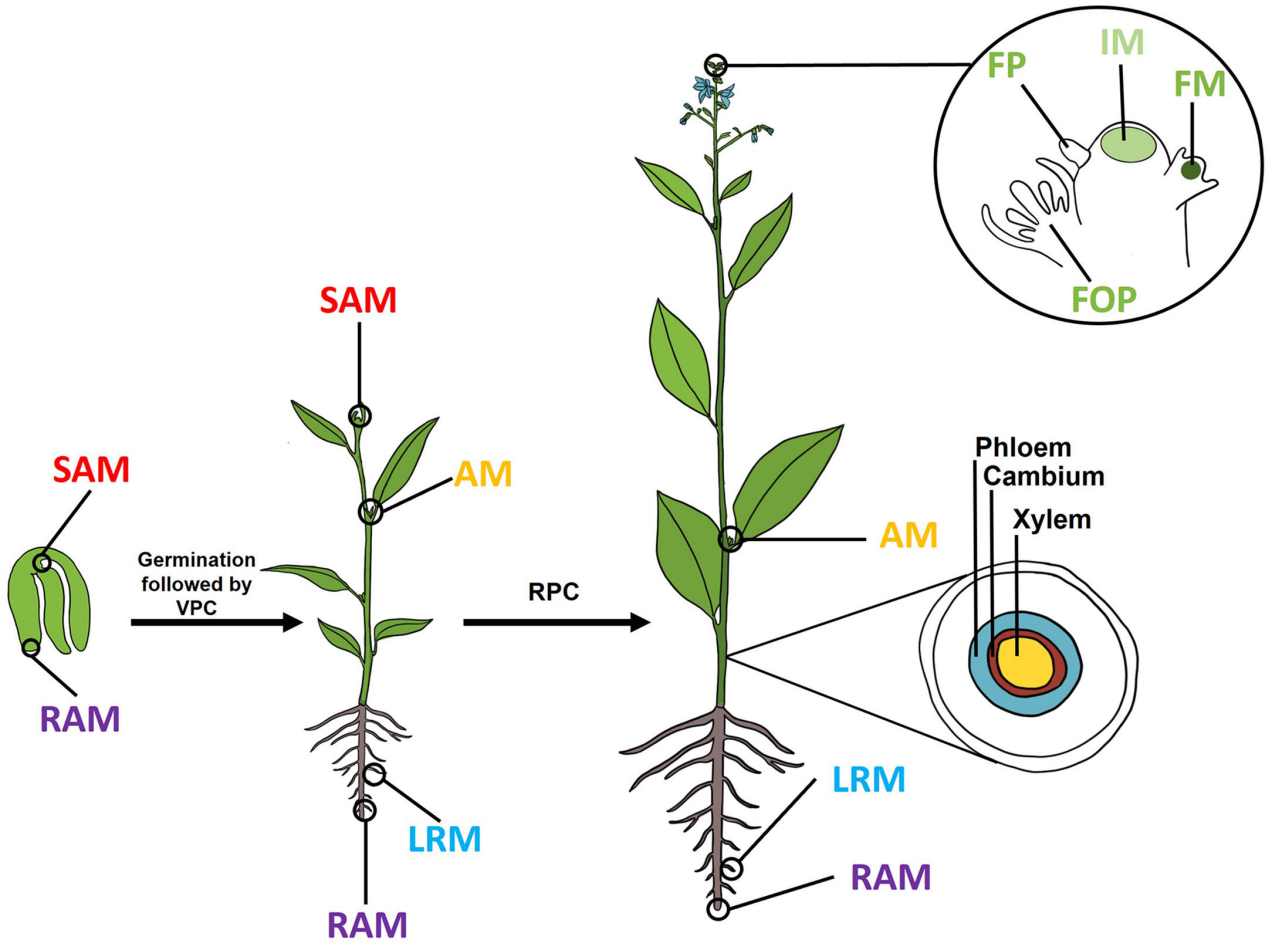
Schematic representation of plant development from germination through reproductive phase transition. The two primary meristems, the shoot apical meristem (SAM) and root apical meristem (RAM), are established during embryogenesis and persist in the mature embryo. Post-embryonically, the SAM produces small groups of cells in leaf axils with meristematic potential, which can reorganize into axillary meristems (AMs). In roots, lateral root (LR) meristems initiate from the pericycle, positioned away from the root tip. Secondary growth is organized by the vascular cambium, a secondary meristem tissue located between the xylem and the phloem. After completion of vegetative phase change (VPC), during reproductive phase change (RPC), the SAM transitions into an inflorescence meristem (IM), which generates floral primordia (FP) that develop into floral meristems (FMs) that generate floral organ primordia (FOP) and ultimately flowers. Key primary meristems are color-coded: SAM (red), RAM (purple), LRM (blue), AM (yellow), IM (light green), FM (dark green). Germination, vegetative phase change and reproductive phase change are indicated by arrows. The inset highlights the shoot apex during flower formation, showing IM and FM positions.

The two primary meristems, the shoot apical meristem (SAM) and the root apical meristem (RAM), are established during embryogenesis, creating the stem cell populations that lay the foundation for all post-embryonic development. The SAM is specified at the apical pole of the embryo, where it gives rise to all aerial tissues, including leaves, stems, and eventually flowers. In parallel, the RAM is initiated at the basal pole of the embryo and gives rise to the primary root system. The SAM and RAM are maintained throughout the plant’s life and are responsible for continuous primary growth. They also provide the developmental framework for the formation of additional primary meristems, such as axillary meristems (AMs), lateral root meristems, and floral meristems (FMs), as well as for secondary meristems, exemplified by the vascular cambium, which are initiated during post-embryonic development (**Figure 1**).

### 2.1 Meristem initiation

The initiation of the SAM during embryogenesis is a highly coordinated process governed by both hormonal cues and key developmental regulators. Among the phytohormones, auxin and cytokinin play pivotal roles in SAM establishment, acting in concert with transcription factors such as WUSCHEL (WUS) and class III homeodomain-leucine zipper (HD-ZIP III) proteins that specify shoot meristem fate (Palovaara et al., 2016). Together, these hormonal and transcriptional networks establish a functional and indeterminate SAM. *WUS*, a central regulator of stem cell homeostasis, is expressed as early as the 16-cell stage of embryogenesis (Mayer et al., 1998). WUS is expressed in the organizing centre (OC) and migrates into overlying stem cells, where it maintains their identity by repressing differentiation (**Table 1**). In response, stem cells produce CLAVATA3 (CLV3), a signalling peptide that, through the CLV receptor complex, restricts *WUS* expression. This WUS–CLV3 negative feedback loop balances stem cell maintenance and OC size (Brand et al., 2000; Schoof et al., 2000). Another critical regulator is SHOOT MERISTEMLESS (STM), which reinforces stem cell identity through a cytokinin-dependent mechanism. STM functions in a feedback loop with *CUP-SHAPED COTYLEDON* (*CUC*) genes: CUC1 and CUC2 activate *STM* expression, while increasing STM levels subsequently repress *CUC* gene activity. This regulatory circuit ensures the proper spatial and temporal initiation of shoot stem cells during embryonic development (Long and Barton, 1998; Takada et al., 2001; Nicolas and Laufs, 2022; Zhang et al., 2023).

**Table 1:** Regulatory networks underlying meristem initiation, maintenance, and fate specification.

	RAM	LRM	SAM	AM	IM	FM
Initiation	Auxin	LBD, Auxin, ARF7/ARF19	Cytokinin	STM, REV, Cytokinin		STM, REV, Cytokinin, Auxin
Stem cell maintenance	WOX5	WOX5	WUS	WUS	WUS	WUS
Feedback loop	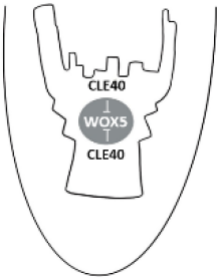 WOX5/CLE40	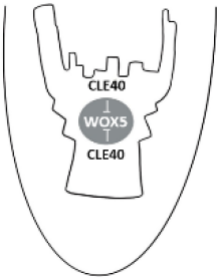 WOX5/CLE40	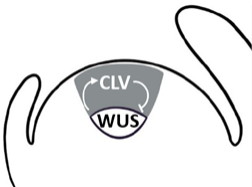 WUS/CLV	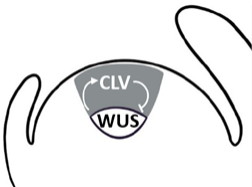 WUS/CLV	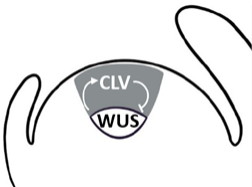 WUS/CLV	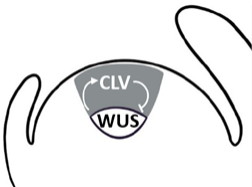 WUS/CLV
Meristem fate	PLTs	PLTs	HD-ZIPIII		FPIs	FMIs

Key regulators are summarized for the root apical meristem (RAM), lateral root meristem (LRM), shoot apical meristem (SAM), axillary meristem (AM), inflorescence meristem (IM), and floral meristem (FM). Initiation signals include auxin, cytokinin, LBD (LATERAL ORGAN BOUNDARIES DOMAIN), and AUXIN RESPONSE FACTOR (ARF) proteins. Stem cell maintenance is mediated by WUSCHEL-RELATED HOMEOBOX 5 (WOX5) in root meristems and WUSCHEL (WUS) in shoot(-derived) meristems. Feedback regulation involves WOX5–CLAVATA3/ESR-related 40 (CLE40) signaling in RAM and LRM, and WUS–CLV3 (CLAVATA3) signaling in SAM, AM, IM, and FM. The IM arises from a developmental transition of the SAM and does not undergo *de novo* initiation. Meristem fate specifies both organ identity (root vs. shoot) and developmental outcome, distinguishing indeterminate inflorescence meristems from determinate floral meristems. Fate regulators include PLETHORA (PLT) proteins (RAM, LRM), class III HOMEODOMAIN–LEUCINE ZIPPER (HD-ZIPIII) proteins (SAM), floral pathway integrators (FPI) (IM), and floral meristem identity (FMI) genes (FM).

The RAM is established even earlier in embryogenesis through coordinated cell divisions and hormonal signalling. Following the asymmetric division of the zygote, the hypophysis, derived from the basal lineage, gives rise to the organizing centre of the RAM, i.e. the quiescent centre (QC) and the columella stem cells. A polar auxin gradient, formed by PIN-FORMED (PIN) transporters, not only establishes a root pole but also initiates a positive-feedback loop with PLETHORA (PLT) family transcription factors. Auxin induces *PLT* expression, and PLTs in turn promote auxin biosynthesis and transport. This self-reinforcing loop creates a stable high-auxin/high-PLT domain that defines root identity and zonation. PIN-mediated auxin transport spatially localizes this activity to the QC and surrounding stem cells, ensuring a finite RAM size (Santuari et al., 2016; Rutten et al., 2021). Concurrently, *WUSCHEL-RELATED HOMEOBOX 5* (*WOX5*) is expressed in the QC, where it maintains quiescence by antagonizing cytokinin-induced cell cycle activation and preserves surrounding stem cell identity by repressing differentiation. SHORTROOT (SHR) and SCARECROW (SCR) further regulate radial patterning and endodermal fate. Together, these factors form an integrated network that specifies and maintains the embryonic RAM (Palovaara et al., 2016; Schwartz et al., 2021).

### 2.2 Meristem activation

After germination, the embryonic SAM and RAM, which have remained dormant (quiescent) in the mature seed, are activated under increased light and photosynthetically-derived sugar, leading to development of the first leaves and root growth by cell division and expansion (Mohammed et al., 2018; López-Juez et al., 2008; Xiong et al., 2013; Nieuwland et al., 2016). In the SAM, these signals are associated with elevated cytokinin activity, which ultimately leads to the reactivation of *WUS* expression, stem cell activation, and the initiation of shoot development (Pfeiffer et al., 2016). Meristem identity is maintained through the WUS-CLV3 negative feedback loop (Han et al., 2020). This regulatory circuit also involves *STM*, which interacts directly with WUS at the protein level. The WUS–STM interaction enhances WUS binding to the *CLV3* promoter, thereby reinforcing *CLV3* expression and contributing to the fine-tuning of stem cell homeostasis (Su et al., 2020). Additionally, STM promotes mitotic competency through the promotion of cytokinin biosynthesis to control the number of meristem cells available for organ formation (Scofield et al., 2013; Lechon et al., 2025).

While the SAM contributes to the main stem, the formation of lateral branches depends on AMs. These structures form post-embryonically from a small group of cells in the leaf axil that retain meristematic potential. These cells proliferate and organize into a structure morphologically and functionally alike to the SAM (Nicolas and Laufs, 2022). A subset of meristem regulators shared with the SAM governs AM initiation. Among these, STM plays a central and early role. Initially, low levels of STM are maintained in the leaf axil by ARABIDOPSIS THALIANA HOMEOBOX GENE1 (ATH1) to preserve meristematic competence (Cao et al., 2020). Subsequently, the HD-ZIP III transcription factor REVOLUTA (REV) directly upregulates *STM* expression, promoting AM initiation (Shi et al., 2016). In contrast, WUS and CLV3, while essential for stem cell regulation in the SAM, are not required for the early stages of AM formation. Activated by cytokinin signalling, their expression appears only after the AM has been established and stem cell activity is initiated, where they function primarily in meristem maintenance, mirroring their role in the SAM (Wang et al., 2017; Xin et al., 2017).

The activation of the embryonic root meristem also initiates a coordinated program of cell division and differentiation that establishes the root meristem and its three major developmental zones: the division zone, elongation zone, and differentiation zone (Verbelen et al., 2006). Centrally positioned within the RAM, the QC constitutes a slow-cycling organizer from which the surrounding division and elongation zones arise. QC identity, marked by *WOX5*, maintains adjacent stem cells and is restrained by CLV3/ENDOSPERM SURROUNDING REGION-related (CLE) peptides, particularly CLE40, which signal through receptors including ARABIDOPSIS CRINKLY4 (ACR4) and CLV1, in a negative feedback loop (**Table 1**; Berckmans et al., 2020; Willoughby and Nimchuk, 2021). Further from the tip, in the differentiation zone, lateral roots arise from pericycle founder cells, a process initiated and patterned by auxin and mediated by key regulators such as LATERAL ORGAN BOUNDARIES DOMAIN (LBD) proteins and AUXIN RESPONSE FACTOR (ARF) proteins ARF7 and ARF19 that activate a new meristematic program (Santos Teixeira and ten Tusscher, 2019; Yalamanchili et al., 2024).

Despite arising in different developmental contexts, plant meristems thus share a remarkably conserved regulatory framework. Core components such as hormonal signals (auxin, cytokinin), stem cell organizers (WUS, WOX5), and feedback loops involving CLE peptides repeatedly occur across shoot, root, and axillary meristems. This high degree of similarity underscores a modular regulatory system that is flexibly deployed to control meristem initiation, competence, and maintenance. Differences between meristems mainly reflect adaptations to their specific roles and timing within the plant life cycle, rather than fundamentally distinct mechanisms (**Table 1**).

Regeneration of meristems in tissue culture closely reflects these endogenous mechanisms, relying on the same hormones and the same suite of developmental regulators. As a result, the targeted application of these regulators has attracted growing interest, particularly for their potential to enhance meristem induction and regeneration in plant transformation systems (Xu et al., 2024; Belaffif et al., 2025; Youngstrom et al., 2025).

In plant tissue culture, meristem formation can occur via somatic embryogenesis (SE), where differentiated cells revert to a totipotent embryonic state, or DNSO, where callus tissues are reprogrammed to form new shoot or root meristems in response to specific hormonal or genetic cues. During SE, differentiated somatic cells return to a totipotent embryonic state under stress and/or hormonal treatments, particularly in response to auxins. From this state, the developmental trajectory resembles that of zygotic embryogenesis, ultimately giving rise to a complete plant (Fehér, 2015). In addition to hormonal treatments, ectopic expression of key transcription factors such as BABY BOOM (BBM), LEAFY COTYLEDON1 (LEC1), LEC2, MYB115, MYB118, and WUS has been shown to induce SE or the formation of embryogenic structures across diverse plant species, highlighting their vital role in reprogramming vegetative cells toward an embryonic identity (Boutilier et al., 2002; Wang et al., 2009; Zheng et al., 2014; Florez et al., 2015; Brand et al., 2019; Xu et al., 2024; Tang et al., 2025).

In contrast, DNSO involves the formation of a pluripotent callus, often at wound sites. In most commonly used protocols, tissue explants are initially cultured on an auxin-rich CIM, followed by transfer to a SIM with elevated cytokinin levels, which stimulates the pluripotent lateral-root-like primordia within the callus to acquire a shoot identity. When incubated on CIM, auxin and wounding signals are perceived, resulting in the production of a pluripotent callus from xylem pole pericycle (XPP) cells that, during normal plant development, give rise to lateral roots (Atta et al., 2009; Sugimoto et al., 2010). Once a pluripotent callus has formed, its further fate in regeneration is decided by auxin and cytokinin. When supplemented with a higher auxin/cytokinin ratio, the callus initiates root regeneration, whereas when supplemented with higher cytokinin/auxin, it initiates shoot regeneration (Skoog and Miller, 1957). A critical step in the latter process is the cytokinin-mediated activation of *WUS* expression. As regeneration proceeds, an initially broad *WUS* expression pattern is reorganized to resemble that of the OC in mature meristems *in planta* (Meng et al., 2017; Zhang et al., 2017).

As with SE, DNSO can also be achieved by directly expressing developmental regulators, such as *WUS* and *STM*, bypassing the need for exogenous hormones. Co-expression of *WUS* and *STM*, or their orthologs, effectively triggers shoot meristem formation and organogenesis across diverse plant species, including *Nicotiana benthamiana* and tomato, thereby enabling regeneration from non-meristematic tissues (Gallois et al., 2002; Maher et al., 2020).

Together, these diverse developmental and regenerative contexts reveal that plant meristems rely on a deeply conserved yet highly flexible regulatory framework. Across tissues, stages, and experimental settings, shoot, root, axillary, and regenerating meristems are specified and maintained through recurring modules of hormonal cues, stem cell-organizer transcription factors, and feedback loops that balance proliferation and differentiation (**Table 1**). This modularity enables plants not only to sustain indeterminate growth throughout their life cycle but also to reconstitute meristems *de novo* during regeneration or *in vitro* culture.

## 3 Meristem fate transitions and the acquisition of floral identity

In angiosperms, the SAM sustains vegetative growth by producing organs such as leaves and stems. The transition to reproductive development typically culminates in the formation of an inflorescence, the flower-bearing shoot. This developmental switch in the SAM, from generating vegetative organs to reproductive structures, requires the meristem to undergo several fate transitions. Both environmental and endogenous cues, including light, ambient temperature, energy status, and developmental age regulate these transitions (Maple et al., 2024).

In most plants, the SAM undergoes two major phase transitions: one within the vegetative phase, known as vegetative phase change (VPC), and another leading to the reproductive phase, referred to as reproductive phase change (RPC) or floral transition. VPC marks the transition from juvenile to adult vegetative identity and is accompanied by coordinated shifts in morphology, physiology, and gene expression that tune development to internal status and environmental cues. In this context, juvenile and adult SAMs differ primarily in their underlying developmental programs: juvenile SAMs generate juvenile leaf traits and, in general, remain reproductively incompetent, whereas adult SAMs produce characteristically adult leaf forms and acquire the competence to initiate flowering (Telfer et al., 1997; Matsoukas, 2014; Zhao et al., 2023).

Subsequently, the adult vegetative SAM transitions into the inflorescence meristem (IM), which generates floral primordia during the reproductive phase of the lifecycle. These floral primordia form a FM, from which the individual floral organs develop (**Figure 1**; Smyth et al., 1990). In *Arabidopsis*, when flower formation is complete, the FM stem cell population terminates, while the IM remains indeterminate and continues to produce new FMs throughout the plant’s reproductive phase.

The development of floral primordia into an FM requires the *de novo* formation of a stem cell niche. In *Arabidopsis*, FMs are considered modified AMs, with the subtending leaf being reduced to a cryptic bract whose growth can be derepressed in some mutant backgrounds (Long and Barton, 2000; Ohno et al., 2004). After primordium initiation, triggered by a local auxin maximum, and an initial growth stage, the floral primordium acquires meristematic features, involving among others STM, WUS and CLV3 (Galvan-Ampudia et al., 2020). Although not expressed in FM founder cells or incipient floral primordia at the flanks of the SAM, *STM* expression is reactivated throughout the apical region of the FM proper when the floral primordium becomes separated from the SAM. Like in AMs, STM plays an important role in maintaining the meristematic ability of the FM (Yang et al., 2023). Around the same time, increased cytokinin signalling is closely followed by the expression of WUS and CLV3, marking the establishment of an OC and a stem cell niche, respectively, and establishing a feedback loop to maintain robust stem cell activity (Long and Barton, 2000).

When all floral organs are initiated, meristem termination is secured by AGAMOUS (AG), which not only specifies stamen and carpel identity but also directly represses WUS by recruiting Polycomb-group proteins (Liu et al., 2011). This repression is reinforced by AG-dependent regulators such as KNUCKLES (KNU), ensuring that the FM ceases stem cell activity once organ initiation is complete (Lenhard et al., 2001; Shang et al., 2021).

The establishment of a meristem alone is not sufficient for flower production as a meristem first needs to acquire a floral fate. Floral fate acquisition is a culmination of factors that follow the earlier-mentioned meristem fate changes. Although it is unclear which specific (combination of) factors are sufficient or necessary to direct the acquisition of flowering fate, the factors that contribute to changes in meristem fate are well-studied.

During VPC, the juvenile SAM matures into an adult SAM, a process that is primarily regulated by the evolutionarily conserved microRNA156 (miR156)/SQUAMOSA PROMOTER BINDING PROTEIN-like (SPL) module. The acquisition of floral competence is governed by a regulatory cascade in which high juvenile levels of miR156 suppress *SPL* expression, thereby delaying both vegetative and reproductive phase transitions. As miR156 levels decline with developmental age, increasing SPL abundance initiates the adult program and promotes miR172 expression, which in turn represses expression of *APETALA2* (*AP2*)-like transcription factors that act as key floral repressors (Wang, 2014; Teotia and Tang, 2015). Although this miR156–SPL–miR172–AP2 module forms a central pathway linking developmental age to flowering, recent work in *Arabidopsis* shows that miR172 can also be activated independently of SPL, indicating that the release of AP2-like repression, and thus floral competence, can proceed even when SPL activity is limited (Zhao et al., 2023). The duration of VPC can take anywhere from a few days to several years, depending on the species and environmental conditions (Pan et al., 2023). Apart from that, endogenous factors, such as plant energy status and phytohormone signalling, control the timing of this (Poethig and Fouracre, 2024). Once competent and exposed to flowering-inducing signals, whether environmental or endogenous, the plant enters the reproductive phase. As a result of RPC, the SAM transitions into an IM that begins to form floral primordia.

**Figure 2:**
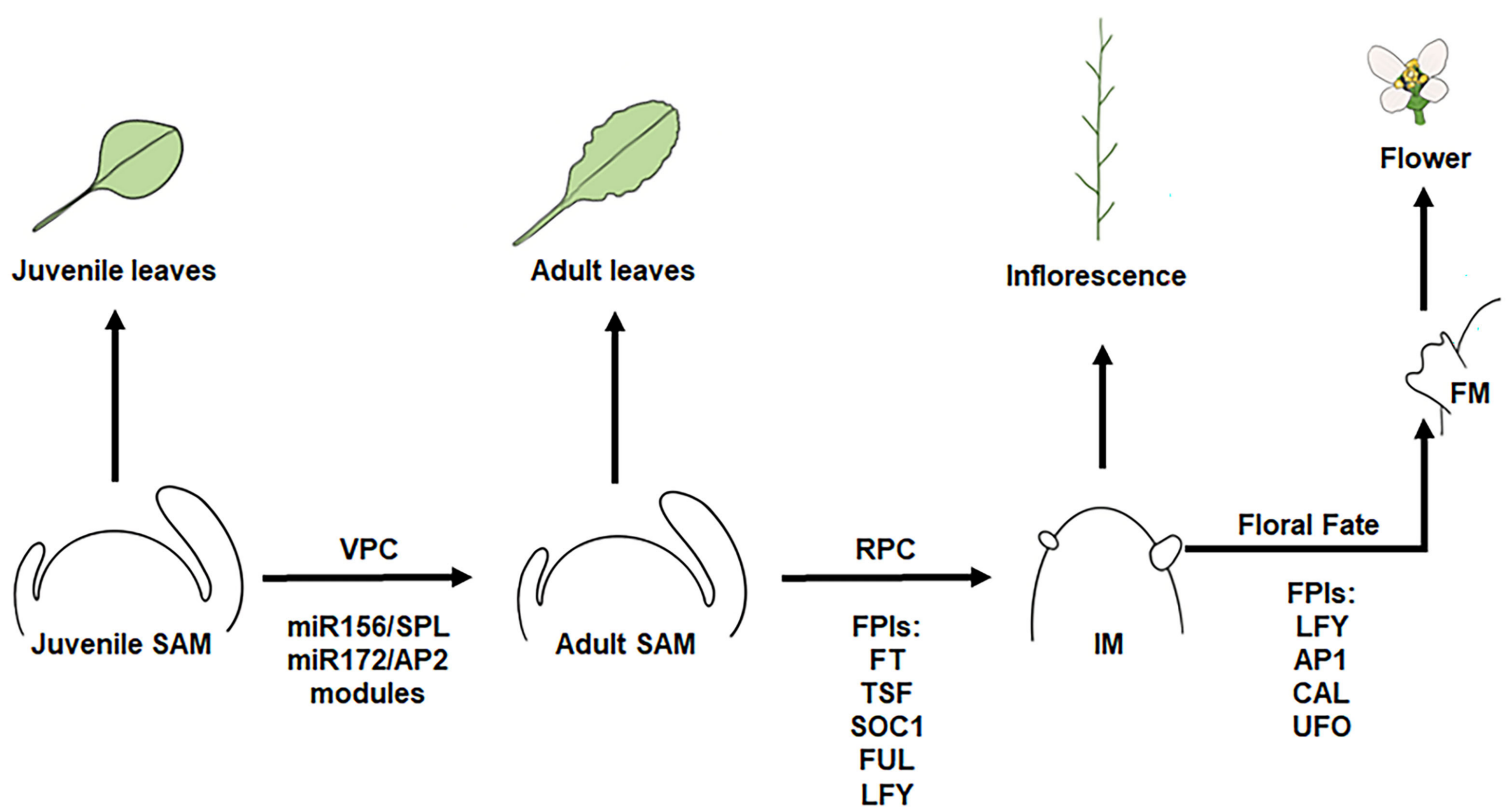
Shoot apical meristem phase transitions and floral fate regulation in Arabidopsis. Sequential transitions of the shoot apical meristem (SAM) during vegetative and reproductive development. Horizontal arrows indicate developmental phase transitions, which are determined by the activity of VPC (vegetative phase change; miR156-SPL-miR172-AP2 modules) and FPI (floral pathway integrator; *FT*, *TSF*, *SOC1*, *FUL*, and *LFY*) gene modules. Upward arrows indicate the products generated by each meristem, which reflect its developmental phase. The juvenile SAM produces juvenile leaves, and the adult SAM produces adult leaves. The inflorescence meristem (IM) gives rise to the main inflorescence, as well as floral primordia at its flanks, which develop into floral meristems (FMs). Floral meristem identity (FMI) genes (*LFY*, *AP1*, *CAL*, *UFO*) commit floral meristems to floral fate to initiate flower formation. VPC and RPC modules specify meristem phase and fate, and this meristem fate determines the identity of the organs that are subsequently produced.

The core regulators of this floral transition are the evolutionarily conserved Floral Pathway Integrators (FPIs), which integrate a broad range of flowering signals. In *Arabidopsis thaliana*, these include FLOWERING LOCUS T (FT), TWIN SISTER OF FT (TSF), SUPPRESSOR OF OVEREXPRESSION OF CONSTANS1 (SOC1), and LEAFY (LFY). The activity of these regulators promotes the transition of the vegetative SAM, which produces leaf primordia, into an IM, which instead produces floral primordia (**Figure 2**). Within the IM, FPIs activate downstream floral meristem identity (FMI) genes, such as *LFY*, *AP1*, *CAULIFLOWER* (*CAL*), and *FRUITFUL* (*FUL*), thereby specifying floral fate and ensuring that the developing primordia give rise to floral meristems rather than leaves (Simpson and Dean, 2002; Wigge et al., 2005; Corbesier et al., 2007).

The promotion of flowering by FPIs FT and TSF, both phosphatidylethanolamine-binding protein (PEBP) family members, occurs through their interaction with FD or its paralogue FD PARALOGUE (FDP). This interaction is mediated by 14-3–3 proteins, a family of highly conserved proteins that can act as adaptor proteins to modulate the function of other proteins, to form the florigen activator complex (FAC) (Taoka et al., 2011; Gao et al., 2025; Romera-Branchat et al., 2025). Once formed at the SAM, the FAC transiently activates *SOC1* and *AP1*, the product of which, together with AGAMOUS-like 24 (AGL24), induce *LFY* expression. LFY and AP1 represent the central FMI regulators: they irreversibly commit primordia to floral fate and initiate the transcriptional networks underlying floral organogenesis. Once LFY and AP1 accumulate, the floral meristem is specified and supported by local auxin signalling.

In contrast, the PEBP-family member TERMINAL FLOWER1 (TFL1) acts as a mobile flowering repressor that also interacts with FD to antagonize FAC activity. In the IM, TFL1 maintains indeterminacy by repressing floral meristem identity genes such as *LFY* and *AP1*, thereby delaying the floral transition. Through this antagonistic action, TFL1 preserves vegetative and inflorescence meristem identity, counteracting FT/TSF signalling and delaying reproductive commitment until environmental and developmental flowering cues become dominant (Périlleux et al., 2019; Cerise et al., 2023).

The initiation of a floral meristem requires the re-establishment of a stem cell niche, marked by the combined expression of *WUS* and *CLV3*. Similar to the SAM, these regulators constitute a minimal genetic circuit that establishes stem cell activity. As floral organ primordia begin to emerge, maintenance of the FM relies on the *WUS*–*CLV3* feedback loop, which sustains the transient stem cell niche until termination is triggered. In addition, floral meristem specification relies on the combined activities of STM, which induces expression of the LFY-cofactor *UNUSUAL FLORAL ORGANS* (*UFO*), and the FT–FD complex, most likely acting together within the FAC (Smith et al., 2011; Roth et al., 2018). Consistent with this, plants with reduced STM function produce arrested flowers that lack carpels and exhibit fewer petals and stamens. Elevated levels of FT and FD can partially rescue these defects, highlighting functional interplay between STM and the florigen pathway during reproductive development (Smith et al., 2011). Ultimately, the FM stem cell population terminates because of the previously mentioned negative autoregulatory mechanism involving *WUS* and AG (Lenhard et al., 2001; Shang et al., 2021). In addition to promoting flowering, the FAC also contributes to flower development by activating genes that define floral organ identity in the inner whorls, including *AG*. In this way, the FAC not only initiates flowering but also indirectly promotes floral meristem determinacy by coupling floral identity specification with meristem termination (Gao et al., 2025; Romera-Branchat et al., 2025).

Taken together, the initiation of floral primordia and their acquisition of floral identity result from the integration of systemic FT-dependent signals and local auxin-driven patterning, converging on *LFY* and *AP1* as master regulators. This is followed by the establishment of a transient stem cell niche and, finally, AG-mediated repression of *WUS*, ensuring proper termination of floral meristem activity and determinate flower development (Chahtane et al., 2023). Given the complexity and rapidly expanding knowledge of flowering regulation, for molecular details readers are referred to the Flowering-Interactive Database (FLOR-ID; (Bouché et al., 2016), which provides a comprehensive, interactive, and continuously updated resource for exploring molecular details of phase transitions and flower formation in Arabidopsis.

## 4 Strategies for orchestrating meristem fate toward direct flower formation in tissue culture

The preceding sections outlined how meristems are initiated and maintained (Section 2) and how they undergo fate transitions toward reproductive development (Section 3). Building on this framework, a central question for *in vitro* flowering is whether these fate transitions can be bypassed or compressed to enable the direct specification of floral meristems from regenerating tissue, which constitutes the basis of DNFO.

FPIs play a conserved role in promoting the switch from vegetative to reproductive development. Ectopic expression of FPIs in diverse species, including monocots and dicots, both annual and perennial, consistently accelerates flowering, both *in planta* and in tissue culture (**Table 2**). However, while FPI overexpression shortens the vegetative phase, it rarely eliminates it: leaves and shoots are typically produced before flowering occurs. *In vitro*, constitutive activation of FPIs can even compromise flower development, leading to malformed or infertile flowers (**Table 2**). These findings underscore both the promise and the limitations of FPI-based strategies: although FPIs clearly accelerate the transition to reproductive development, their expression alone is insufficient to orchestrate the complex developmental program required for the formation of complete and fertile flowers.

**Table 2:** Accelerated flowering (in planta) and/or flower formation in vitro following genetic manipulation of floral pathway integrators and meristem identity genes.

Species	Approach	FPI/FMI	*In planta*/ *in vitro*	Remark	Reference
*Arabidopsis*	*p35S::LFY*	FPI/FMI	*In planta*		(Weigel and Nilsson, 1995)
Aspen	*p35S::LFY*	FPI/FMI	*In vitro*		
*Arabidopsis*	*pLFY::LFY*	FPI/FMI	*In planta*	Additional copies of wild-type *LFY* alleles	(Blázquez et al., 1997)
*Arabidopsis*	*p35S::LFY:VP16*	FPI/FMI	*In planta*	Most organs are tipped with stigmatic tissue	(Parcy et al., 1998)
	*p35S::LFY*	FPI/FMI	*In planta*		
	*p35S::LFY p35S::UFO*	FPI/FMI	*In planta*	Seedlings were growth-arrested and did not form any mature leaves	
*Arabidopsis*	*p35S::FT*	FPI	*In planta*		(Kardailsky et al., 1999)
	*p35S::LFY*	FPI/FMI	*In planta*		
	*p35S::LFY p35S::FT*	FMI/FPI	*In planta*		
	*p35S::AP1*	FMI	*In planta*		
	*p35S::FT p35S::AP1*	FPI/FMI	*In planta*		
*Arabidopsis*	*p35S::FT*	FPI	*In planta*		(Kobayashi et al., 1999)
	*p35S::LFY*	FPI/FMI	*In planta*		
	*p35S::FT* × *p35S::LFY*	FPI/FMI	*In planta*	SAM was terminated and transformed into a single terminal flower	
Tobacco	*p35S::LFY*	FPI/FPI	*In planta*	Aberrant terminal flower	(Ahearn et al., 2001)
Tobacco	*p35S::NFL*	FMI	*In planta*	Flowers exhibited supernumerary floral organs	
*Arabidopsis*	p35S::*MdMADS5*	FMI	*In planta*	MdMADS5 = AP1 orthologue	(Kotoda et al., 2002)
*Arabidopsis*	*p35S::CDM111*	FMI	*In planta*	CDM111 = AP1 orthologue	(Shchennikova et al., 2004)
*Arabidopsis*	*p35S::LFY:GR* in regenerating root explants	FPI/FMI	*In vitro*		(Wagner et al., 2004)
	*p35S::LFY* in regenerating root explants	FPI/FMI	*In vitro*		
Trifoliate orange	*p35S::CiFT*	FPI	*In planta*		(Endo et al., 2005)
*Arabidopsis*	*p35S::TSF*; *p35S::LFY*	FPI	*In planta*		(Yamaguchi et al., 2005)
*Arabidopsis*	*p35S::TSF*	FPI	*In planta*		
Tomato	*p35S::SFT*	FPI	*In planta*		(Lifschitz et al., 2006)
*Arabidopsis*	*pBLS::SFT*	FPI	*In planta*	*BLS* promoter drives expression in leaf primordia and young leaves	
Tobacco	*p35S::SFT*	FPI	*In planta*		
Apple	*p35S::BpMADS4 *	FPI	*In vitro*	MADS4 = FUL orthologue from *Betula pendula*	(Flachowsky et al., 2007)
*Arabidopsis*	*p35S*::*AGL20*	FPI	*In planta*	AGL20 = SOC1; Abnormal flowers: sepaloid petals, elongated non-self-pollinated carpels	(Borner et al., 2008)
Tobacco	p35S::*MADSA*	FMI	*In planta*	MADSA = AGL20/SOC1 orthologue	
*Arabidopsis*	*p35S::DOT*	FMI	*In planta*		(Souer et al., 2008)
*Petunia*	*p35S::DOT*	FMI	*In planta*		
	*p35S::DOT p35S::ALF*	FPI/FMI	*In planta*	Growth arrested	
	*p35S::UFO p35S::LFY*	FPI/FMI	*In planta*	Growth arrested	
*Arabidopsis*	*p35S::BvFT2*	FPI	*In planta*		(Pin et al., 2010)
*Beta vulgaris ssp. vulgaris*	*p35S::BvFT2*	FPI	*In planta*	Flowers are larger than in WT	
*Arabidopsis*	*p9N-35S::MdFT1 *	FPI	*In planta*		(Tränkner et al., 2010)
	*p9N-Suc2::MdFT1 *	FPI	*In planta*		
Apple	*p9N-35S::MdFT1*	FPI	*In vitro*	First flowers during *in vitro* cultivation	
*Populus*	*p9N-35S::MdFT1*	FPI	*In vitro*	*In vitro* flowers; modified perianth	
	*35S::AtFT*	FPI	*In vitro*	Single flowers and some catkins	
*Populus*	*pHSP::FT*	FPI	*In planta*	Abnormal flowers: variation in development and morphology among floral structures produced	(Zhang et al., 2010)
	*pHSP::FT1*	FPI	*In planta*	Abnormal flowers: variation in development and morphology among floral structures produced	
	*pHSP::FT2*	FPI	*In planta*	Abnormal flowers: variation in development and morphology among floral structures produced	
*Medicago truncatula*	*p35S::FTa1*	FPI	*In planta*		(Laurie et al., 2011)
Chrysanthemum	*p35S::CDM111*	FMI	*In planta*	CDM111 = AP1 orthologue	(Shulga et al., 2011)
European pear	RNAi of *PcTFL1-1* and *PcTFL1-2*	FPI	*In planta*	Flowers form additional sepals	(Freiman et al., 2012)
*Arabidopsis*	*p35S*::*FaSOC1*	FPI	*In planta*		(Lei et al., 2013)
*Arabidopsis*	*p35S::UFO-VP16*	FMI	*In planta*		(Risseeuw et al., 2013)
*Brassica napus*	*p35S::UFO:VP16*	FMI	*In planta*		
Tobacco	*p35S::UFO:VP16*	FMI	*In planta*		
Tobacco	*p35S::VcFT*	FPI	*In planta*		(Song et al., 2013)
Bluebberry	*p35S::VcFT*	FPI	*In vitro*	In vitro flowering, but flowers did not develop into fruits conditions	
Blueberry	*p35S::VcFT*	FPI	*In planta*	*VcFT* overexpression resulted in a lower transformation frequency; transgenes formed fewer branches and new shoots	(Gao et al., 2016)
*Arabidopsis*	*p35S::PvSOC1*	FPI	*In planta*		(Liu et al., 2016a)
Rice	*p35S::PvSOC1*	FPI	*In planta*	Overexpression results in shorter panicles and reduced fertility	
*Arabidopsis*	*p35S::PvMADS56*	FPI	*In planta*	AGL20/SOC1 orthologue; reduced or no flower fertitlity	(Liu et al., 2016b)
Kiwifruit	*p35S::AcFT*	FPI	*In vitro*		(Moss et al., 2018)
Soybean	*p35S::GmAP1a*:*3Flag*	FMI	*In planta*		(Chen et al., 2020)
*Arabidopsis*	*p35S::TcFT*	FPI	*In planta*		(Prewitt et al., 2021)
*Theobroma cacao*	*p35S::AtFT*	FPI	*In vitro*		
*Arabidopsis*	*p35S::MiCAL1*	FMI	*In planta*		(Xie et al., 2023)
	*p35S::MiCAL2*	FMI	*In planta*		

Studies reporting genetic interventions targeting key flowering regulators. Ectopic expression of floral pathway integrators (FPIs) or floral meristem identity (FMI) genes, or silencing of their repressors, has been examined across diverse species, including monocots and dicots, as well as annual and perennial plants, in both *in planta* and *in vitro* systems. These interventions frequently result in accelerated flowering, but constitutive activation of FPIs or FMIs may also compromise floral development, leading to malformed or infertile flowers.

FMI genes such as *LFY* and *AP1* provide a further step toward direct flowering. *LFY* acts as both an FPI and FMI gene, functioning as a pioneer transcription factor that opens chromatin and activates *AP1* (Jin et al., 2021). This highly conserved, plant-specific transcription factor is pivotal for both floral fate determination and subsequent floral patterning (Moyroud et al., 2009; Gao et al., 2019). Its importance for floral fate commitment is underscored by the severe developmental defects in *lfy* mutants across species, and by gain-of-function experiments where *LFY* or *LFY* orthologs, such as *FALSIFLORA* (*FA*) or *NICOTIANA FLO*/*LFY* (*NFL1*), accelerate flowering (Coen et al., 1990; Weigel and Nilsson, 1995; Molinero-Rosales et al., 1999; Ahearn et al., 2001; Bomblies et al., 2003; Siriwardana and Lamb, 2012; Quevedo-Colmena et al., 2025). Despite this accelerated flowering, these plants generally still undergo a brief vegetative phase. A similar pattern of accelerated, but sequential, flowering occurs in plants ectopically expressing other FMIs, suggesting that a meristem first has to acquire competence to respond to the activity of these genes (Kotoda et al., 2002; Shulga et al., 2011; Zhou et al., 2019; Chen et al., 2020; Xie et al., 2023). Even combined overexpression of FT and LFY generally still leads to sequential transition, with vegetative growth preceding flower initiation, although direct flowering has occasionally been reported (Kardailsky et al., 1999; Kobayashi et al., 1999). In contrast, under *in vitro* conditions, LFY can bypass the typical vegetative phase and directly induce floral fate. For example, in *Arabidopsis* root explants expressing the post-translationally regulated *35S:LFY-GR* construct, *LFY* induction during shoot regeneration triggered the direct formation of flowers or floral organs without prior development of rosette leaves (Wagner et al., 2004). Similarly, co-expression of *LFY* with *WUS* in roots triggers floral organ formation from WUS-induced meristems. However, these organs are disorganized and show random combinations of sepals, stamens, and carpels (**Table 2**; Gallois et al., 2004).

This activity may depend on the presence of cofactors, such as UFO (Weigel and Nilsson, 1995; Chae et al., 2008; Sasaki et al., 2012; Rieu et al., 2023b). *UFO* encodes an F-box protein that partners with LFY to regulate floral identity targets via LFY–UFO Binding Sites (LUBS). While UFO can act as an F-box protein in ubiquitination pathways, this activity is largely dispensable for its main role with LFY. Instead, UFO primarily functions as a transcriptional cofactor, enabling selective activation of a subset of target genes important for flower and inflorescence development (Rieu et al., 2023b). In this way, UFO alters LFY’s DNA-binding specificity, expanding its regulatory versatility. As UFO has a weak affinity for DNA and requires LFY for all known functions, it is thought that UFO does not regulate any genes by itself in *Arabidopsis* and depends on the formation of a complex with LFY to regulate gene expression (Lee et al., 1997; Risseeuw et al., 2013; Rieu et al., 2023b). This dependence on LFY is emphasized in *ufo* loss- and gain-of-function mutants, where the *ufo* phenotype is completely masked in a *lfy* background (Levin and Meyerowitz, 1995; Wilkinson and Haughn, 1995; Lee et al., 1997). Notably, this strict interdependence appears to be evolutionarily conserved. Although LFY generally plays a broader role, spatially restricted expression of UFO enables LFY to act more selectively in defined developmental regions across angiosperms (Rieu et al., 2023a).

Beyond their well-established roles in floral meristem identity and floral patterning, both LFY and UFO have been suggested to display features characteristic of developmental regulators with meristematic potential. In *Arabidopsis*, LFY has been shown to promote AM activity (Chahtane et al., 2013). In legumes such as pea, *Lotus*, and alfalfa, LFY orthologs promote the transient meristematic state in developing leaf primordia required for compound leaf formation, while in grasses, including rice and maize, LFY orthologs contribute to sustained meristematic growth in inflorescences (Moyroud et al., 2009). Likewise, monocot UFO orthologs such as APO1 in rice, WAPO1 in wheat, and ZmAPO1–9 in maize extend their functions to regulate meristem proliferation, including suppression of vegetative growth and promotion of inflorescence meristem proliferation (Wittern et al.,2022; Ikeda-kawakatsu et al., 2009; Ikeda et al.,2007; Jiang et al., 2023). Consistent with these observations, expression of a gain-of-function UFO fusion protein (UFO–VP16; a translational fusion of UFO and a transcriptional activation domain) in *Arabidopsis* triggers LFY-dependent formation of ectopic meristems on leaves that develop into flowers, bracts, and inflorescences (Risseeuw et al., 2013). Collectively, these findings highlight LFY and UFO as promising targets for direct flowering induction in tissue culture, capable of both triggering floral fate and reprogramming meristem behaviour.

Although LFY and UFO clearly hold potential as tools for direct floral induction, their deployment in tissue culture requires caution. As outlined, successful floral induction depends not only on the initiation and maintenance of a meristematic state but also on its timely transition toward reproductive fate. Constitutive or ectopic expression of *LFY* or *UFO* can disrupt this balance, frequently producing pleiotropic effects such as floral organ abnormalities and premature termination of the meristem through depletion of the stem cell pool. Plants constitutively overexpressing *UFO* or its orthologs frequently exhibit floral abnormalities, including increased numbers of floral organs, organ chimeras, missing carpels, and malformed gynoecia (Souer et al., 2008). Likewise, combined ectopic overexpression of *LFY* and *UFO*, or of their *Petunia* orthologs *ABERRANT LEAF AND FLOWER* (*ALF*) and *DOUBLE TOP* (*DOT*), leads to severe developmental defects, resulting in growth-arrested seedlings in both *Arabidopsis* and *Petunia* (Parcy et al., 1998; Souer et al., 2008).

Meristem arrest upon combined overexpression of *LFY* and *UFO* most likely arises from premature specification of floral fate throughout the IM. This overrides the indeterminate program, in part through repression of *TFL1*, a key antagonist of LFY required to maintain indeterminacy (Gustafson-Brown et al., 1994; Liljegren et al., 1999; Hanano and Goto, 2011; Goslin et al., 2017; Serrano-Mislata et al., 2017). In addition, ectopic co-expression of *LFY* and *UFO* might trigger precocious and spatially expanded activation of *AG*, which normally terminates floral meristem activity by repressing *WUS* (Sun and Ito, 2015). When *AG* is activated too early or in inappropriate domains, *WUS* is prematurely silenced, causing early exhaustion of the stem cell population. As a result, the floral meristem ceases activity before sufficient organ primordia are produced, yielding incomplete or abnormal flowers. Consistent with this view, single-meristem transcriptome analysis revealed that the floral transition in tomato proceeds through discrete temporal states, with tightly ordered waves of gene expression coordinating meristem maintenance and floral fate acquisition (Meir et al., 2021). Their findings emphasize that precise timing of regulators such as LFY and UFO is essential, and that premature or prolonged expression destabilizes meristem function. Accordingly, inducible or transient delivery of LFY and UFO, via regulated promoters or cell-penetrating peptides (Sant’Ana et al., 2020), may better mimic their natural expression dynamics, enabling floral induction without causing premature meristem termination.

## 5 Prospects in direct flower formation from regenerating meristems

Developing systems for direct flower formation from callus-derived meristems addresses a key practical challenge: accelerating breeding and flower production in long-generation crops while reducing labour, space, and time requirements. The convergence of pathways controlling meristem initiation and floral fate acquisition points toward new opportunities to design systems that enable DNFO, i.e., direct flower formation from regenerating meristems. Advances in our understanding of meristem competence and maintenance have enabled strategies to induce meristems via DR genes. Complementary studies on meristem fate-change regulators, such as FPI and FMI, demonstrate that developmental fate transitions can be accelerated or redirected.

However, neither FPIs nor LFY alone are sufficient to induce complete flowers directly from callus or regenerating tissues. Coordinated *LFY* and *UFO* expression may combine aspects of both DR and FMI activity. This strategy exemplifies the DNFO approach, where meristem initiation and floral fate acquisition are coordinated to induce floral meristems directly from regenerating tissues. Whether UFO alone is sufficient to generate fertile flowers remains to be established, but this possibility highlights the convergence between developmental reprogramming and floral induction research. From an applied perspective, co-expression of *LFY* and *UFO* could enable: (i) bypassing intermediate vegetative development, allowing explants to produce flowers directly; (ii) synchronizing meristem initiation with floral fate commitment, reducing developmental variability; (iii) shortening breeding cycles by decoupling flower formation from endogenous cues.

A major challenge in achieving direct flower formation through co-expression of *LFY* and *UFO* is premature meristem termination during floral induction. Future work should explore inducible, tissue-specific, or synthetic regulatory systems to coordinate *LFY*–*UFO* activity during regeneration. Additionally, the dependence of LFY on UFO for activating target genes appears to vary across species. To harness the full flowering potential of the LFY–UFO complex, a deeper understanding of their co-regulated targets and interaction dynamics is needed. Such knowledge will be essential for designing expression strategies tailored to species-specific regulatory contexts. Insights into downstream UFO regulation remain limited to a few model species (*Arabidopsis*, tomato, *Petunia*), and the conservation of the LFY–UFO network across plant taxa is largely unknown. Comparative studies could clarify how floral meristem formation is organized in different species and why certain synthetic combinations of LFY and UFO (e.g., LFY–GR and UFO–VP16 in *Arabidopsis*) trigger direct flowering phenotypes.

With these advances, the rational design of systems that induce meristems from callus and then directly specify floral fate could evolve from a conceptual vision into a practical tool for developmental reprogramming and crop improvement. Especially for long-generation crops, skipping vegetative stages could significantly accelerate breeding programs. Furthermore, this technology allows synchronized and predictable flowering, reduces resource and labour requirements, and enables rapid evaluation of flower-specific traits, making it a versatile platform for research on floral meristem specification and regulatory networks. It also has potential industrial applications, including ornamental production, hybrid generation, and synthesis of flower-derived compounds. Moreover, such direct flower formation systems may enable entirely novel production modalities, including “cultured fruit” production without the need to grow full plants, underscoring the potential of this technology not only for breeding and horticulture, but also for fundamentally rethinking how we produce plant-derived goods (van der Zee et al., 2025). Overall, further research on the mechanisms that drive flower formation and on how to use this knowledge to induce flowering artificially can provide a faster, more controlled, and resource-efficient route from tissue to flowers, with clear scientific, economic, and industrial significance.
